# Beyond job security and money: driving factors of motivation for government doctors in India

**DOI:** 10.1186/1478-4491-12-12

**Published:** 2014-02-21

**Authors:** Bhaskar Purohit, Tathagata Bandyopadhyay

**Affiliations:** 1 Indian Institute of Public Health, Gandhinagar, Sardar Patel Institute Campus, Drive in Road, Thaltej, Ahmedabad 380054, Gujarat, India; 2 Indian Institute of Managemant (IIM), Vastrapur, Ahmedabad, Ahmedabad 380015, Gujarat, India

## Abstract

**Background:**

Despite many efforts from government to address the shortage of medical officers (MOs) in rural areas, rural health centres continue to suffer from severe shortage of MOs. Lack of motivation to join and continue service in rural areas is a major reason for such shortage. In the present study, we aimed to assess and rank the driving factors of motivation important for in-service MOs in their current job.

**Methods:**

The study participants included ninety two in-service government MOs from three states in India. The study participants were required to rank 14 factors of motivation important for them in their current job. The factors for the study were selected using Herzberg’s two-factor theory of motivation and the data were collected using an instrument that has an established reliability and validity. Test of Kendall’s coefficient of concordance (W) was carried out to assess the agreement in ranks assigned by participants to various motivation factors. Next, we studied the distributions of ranks of different motivating factors using standard descriptive statistics and box plots, which gave us interesting insights into the strength of agreement of the MOs in assigning ranks to various factors. And finally to assess whether MOs are more intrinsically motivated or extrinsically motivated, we used Kolmogorov-Smirnov test.

**Results:**

The (W) test indicated statistically significant (*P* < 0.01) agreement of the participants in assigning ranks. The Kolmogorov-Smirnov test indicated that from policy perspectives, MOs place significantly more motivational importance to intrinsic factors than to extrinsic factors. The study results indicate that job security was the most important factor related to motivation, closely followed by interesting work and respect and recognition. Among the top five preferred factors, three were intrinsic factors indicating a great importance given by MOs to factors beyond money and job security.

**Conclusion:**

To address the issue of motivation, the health departments need to pay close attention to devising management strategies that address not only extrinsic but also intrinsic factors of motivation. The study results may be useful to understand the complicated issue of work motivation and can give some useful insights to design comprehensive management strategies that are based on motivational needs of MOs.

## Introduction

According to the World Health Report (WHR) [1], there are at least 57 countries in the world with a critical shortage of health-care workers. The WHR defines *critical shortage* as a health-care worker to population density ratio below a critical threshold of 2.3 per 1000 population. Addressing the issue of shortage is important because the health-care systems in countries with shortages of health-care providers are unable to offer even the most basic health services to their citizens [1]. The WHR defines *basic health* as 80% immunization coverage and 80% skilled birth attendance at delivery. Empirical evidence indicates that an adequate health-care workforce is essential to achieve a minimum level of health indicators [1,2]. An inadequate number of health-care workers is associated with poor quality of health services, especially in rural areas [3]. Therefore, an effective health-care system needs to have an adequate-sized, well-motivated, skilled health-care workforce [4]. Although some of countries have made remarkable progress in addressing their health-care workforce shortages and imbalances, a lot needs to be done in a country such as India.

Within the health-care workforce, service providers (particularly doctors) or medical officers (MOs) are a key to effective management and supervision of health-care services, especially in rural areas. However, there is a significant shortage of MOs in the Indian public health system. Estimates indicate that the public health system employs at most 10% of total MOs in India [5]. India also has a considerable discrepancy in where the MOs are placed and distributed. Such differences exist between the states as well as within the states. For example, the doctor to population ratio is as high as one per 470 people in the state of Delhi to as low as one per 15 547 in the state of Haryana. Furthermore, there is a clear disparity between the distributions, which often favour urban areas. For instance, the ratio of rural doctors to rural population is far less or poor than the ratio of total doctors to total population [5]. The overall country figures for India also suggest that the vacancy rate of MOs is nearly 21% at primary health centres (PHCs) and 42% for specialists at community health centres (CHCs) [6]. A PHC covers a population of 20 000 in hilly, tribal or difficult areas and 30 000 population in areas on the plains with four to six indoor observation beds. It acts as a referral unit for six subcentres and refers cases to CHCs (30-bed hospitals) and higher-order public hospitals located at the subdistrict and district levels. *Specialists* herein refers to surgeons, obstetricians/gynaecologists, physicians and paediatricians. A CHC is a 30-bed hospital that constitutes the secondary level of health care and provides referrals as well as specialist health care to rural populations at the block level. It caters to 80 000 to 1 200 000 population.

There are two broad strategies for addressing the shortage of MOs. One is to attract MOs to join the rural health-care services (which, according to Herzberg theory, are called hygiene factors) and the other is to retain the existing and the newly recruited MOs and manage their performance (which, according to Herzberg theory, are called motivating factors). We discuss the Herzberg theory of motivation later in this article. The response of many Indian states to address the shortage has focused more on the first strategy: attracting and recruiting MOs to rural areas. Such a response is based upon two assumptions: (1) Most MOs prefer to work in urban areas and a have high preference to pursue postgraduate degrees (PGs), and (2) money is an important factor in attracting MOs to serve in rural areas. These assumptions have led to designing strategies that take advantage of the fact that MOs tend to pursue PG degrees and that they have a high preference to work in urban areas. Hence, strategies implemented by Indian states fall under regulatory mechanisms such as compulsory rural service, recruitment of MOs on a contractual basis and providing monetary and nonmonetary benefits [7-10]. Recent studies indicate that compulsory rural service cannot be an effective stand-alone measure to attract and retain service providers in a health system [11]. Despite efforts in this direction, many health systems fail to attract and retain health-care providers in rural areas. Thus studies to understand the factors that motivate MOs to join and, more importantly, to continue service in government health centres in rural areas have recently received more attention [12,13]. Attention to motivation is important because factors that influence the performance of health-care workers are a function of motivation [14]. Motivation and job satisfaction have been identified as important determinants of the retention and performance of health-care workers [14-17]. Also, work motivation has been found to be a major determinant of health sector performance [18].

In India, despite the government’s efforts to increase resources for the health-care sector, health care indicators remain very low and the quality of health-care services delivered by the government is generally poor [19]. Lack of motivation among health-care workers engaged in the public sector results in high rates of absenteeism, which is approximately 43% [18]. On top of that, huge migration of Indian doctors to other countries adds to the problem of the shortage of health-care professionals [20,21]. From the policymakers’ perspective, the key challenges are to attract, retain and motivate doctors to enhance the effectiveness and efficiency of the existing health-care system.

Hence, in the current study, we aimed to assess the most important factors of motivation for in-service MOs in their current jobs and to rank these factors from top to bottom based on MOs’ perceived importance of each factor. The 14 factors of motivation for the study were selected using an appropriate instrument that captured seven intrinsic and seven extrinsic factors of motivation based on the Herzberg two-factor theory, which is discussed in detail in the next section. Although the factors chosen for this study are based on Herzberg’s two-factor theory, the study is not completely based on it. This aspect is discussed in the next section as well.

In the present study, we used a ranking method to assess motivation over the discrete choice experiments (DCEs) because the ranking method has several advantages. For example, the data for the current study reflect revealed preferences (RPs), which represent the actual choices made by the respondents, in contrast to the stated preferences (SPs), which represent choices in a hypothetical context [22]. Hence, RP data are more reliable. We also carried out the analysis based on the ranks instead of converting it into multiple binary observations and then using discrete choice models [22,23]. The reasons for this are twofold. First, use of discrete choice models would make sense if, along with the data on ranking preferences, we had data on the psychographic and demographic characteristics of the MOs. Second, discrete choice formulation is more useful for SP data to control for its unreliability [24]. Because we did not analyse the data on the basis of psychographic or demographic characteristics or on SPs, we conducted a study based on ranks using the Herzberg two-factor theory of motivation.

As the current study was based on ranking exercise, we first assessed whether the assigned ranks by the respondents showed any statistically significant agreement. Once this factor was assessed, the next step carried out was to rank the factors and study their distributions using box plots as well as standard descriptive statistics.

## What is motivation?

Different researchers have described motivation differently. According to Hitt *et al.*, “Motivation refers to the forces coming from within the person that account, in part, for the wilful direction, intensity and persistence of the person’s efforts towards achieving specific goals that are not due to ability or to environmental demands” [25]. According to Luthans, “Motivation is the process that arouses, energizes, directs, and sustains behaviour and performance” [26]. According to Robbins *et al.*, “Motivation is the process that accounts for an individual’s intensity, direction and persistence of effort towards attaining a goal” [27]. Research indicates that different individuals work for different reasons and have different motivations to work [28].

Work motivation has been described as “psychological processes that direct, energize, and maintain action toward a job, task, role, or project” [29,30]. Just as different individuals have different motivations to be engaged in a particular type of work, the same is true for motivation within that type of work. For example, some individuals are motivated by benefits such as an adequate salary, job security, good working conditions and organizational policies. These motivations are called *external motivational needs*. Some individuals are motivated by factors such as achievement, growth, advancement, respect and recognition, independence, and responsibility. These motivations are called *internal motivational needs*. In the present study, we focus on assessing whether MOs from the three states are externally motivated only or are internally motivated as well.

## Theory of motivation relevant to our research

There are many theories of motivation. In this article, we discuss only Herzberg’s two-factor theory of motivation because we used the motivation factors based on this theory in the present study [31-33]. Herzberg’s two-factor theory of motivation was used for the study for several reasons. We used an instrument for data collection with established reliability and validity. This instrument is based on the Herzberg theory of motivation. We feel that the Herzberg theory is very relevant to health-care settings in India. The literature published on motivation in India covers many factors that have been proposed by Herzberg. On the basis of a pretest of the selected instrument, however, we made one modification to the study theory. This is discussed in more detail later in this section.

According to Herzberg’s two-factor theory of motivation, the factors that cause job satisfaction at work (which Herzberg calls motivators/intrinsic factors/job content factors) are different from the ones that cause job dissatisfaction (which he calls hygiene/extrinsic/job context factors). The term *hygiene*, as used by Herzberg, is very different from the one used in the health care field. Herzberg uses *hygiene* to describe the job factors which are considered to be just the maintenance factors that are important to avoid dissatisfaction with work, but do not necessarily provide satisfaction or positive motivation. In his theory, he draws a qualitative difference between hygiene and motivating factors. For example, on the one hand, motivators or intrinsic factors such as recognition, if met in a job, produce job satisfaction. On the other hand, hygiene/extrinsic/job context factors, such as job security, if not met, produce job dissatisfaction. If hygiene factors are met, however, they prevent only job dissatisfaction. According to this theory, the factors causing satisfaction are different from those causing dissatisfaction, hence the two feelings should not be treated as opposites of one another. The main distinction that Herzberg makes between hygiene and motivators is that hygiene factors are not directly related to work, so he calls them ‘job context factors’, whereas motivators are directly related to work, so he calls them ‘job content factors’. According to Herzberg, motivators contribute to people’s satisfaction (and development) in their jobs [34].

Although in the present study we used some of the most important factors proposed by Herzberg, they are slightly different from those described in the original theory. For example, we did not ask the respondents to differentiate between hygiene and motivators. We did not do so because, during the pretest of the instrument, we discovered that the respondents found it difficult to differentiate between hygiene and motivators and that sometimes they treated the two terms merely as the opposite of one another. Hence, to address these issues, we included 14 factors (seven hygiene and seven motivators) and mixed them together in the study instrument without asking participants to differentiate between the two. The study participants were simply asked to rank these factors according to the perceived importance they place on each factor that motivates them in their current job.

When we discuss the study results, however, we categorize each factor as either intrinsic or extrinsic because of the general understanding of the theory that hygiene factors are the extrinsic factors that are important to attract individuals to a job and continue in it. Intrinsic factors are the ones that concern individual performance and development within a job.

## Methods

### Ethical approval

As the study did not involve any drug trial or invasive procedures and was done in an educational setting, no ethical clearance from institutional ethical review committee was required. The identities of the respondents is kept confidential. Verbal consent was obtained from the study participants.

### Study design and study respondents

The current study was cross-sectional and was aimed at assessing the driving factors of motivation for in-service MOs in three states in India. The study respondents included 92 in-service MOs who attended a 1-year postgraduate diploma programme in public health management (PGDPHM) in the states of Gujarat, Madhya Pradesh (MP) and Orissa. In the total sample, 52 MOs were from Gujarat, 22 were from MP and 18 were from Orissa. The study respondents worked at different health-care facilities, including PHCs, CHCs and DHs, and represented more than 25 districts in the three states in India. DHs are public hospitals that cater to the health-care needs of the entire district and provide mainly tertiary care. In total, there were 57 MOs from PHCs, 28 from CHCs and seven from DHs (see Table 1).

**Table 1:** Distribution of respondents according to Indian state and type of health-care centre

**State**	**PHCs**	**CHCs**	**DHs**	**Total**
Gujarat	30	18	4	52
Madhya Pradesh	12	7	3	22
Orissa	15	3	0	18
Total	57	28	7	92

CHC community health centre, DH district hospital, PHC primary health centre.

The majority of the PGDPHM programme participants were in-service MOs working at various government health-care centres in the three Indian states. Recognizing the importance of public health management training, various Indian states have made a commitment to send some of their MOs who are already working to attend a 1-year training programme called the PGDPHM. The participants in the PGDPHM were no different from the MOs working with the public health system, except they had an opportunity to attend the PGDPHM programme based on certain selection indicators. PGDPHM programme participants returned to their work after completing the diploma program. The data collection from the study participants was done at the beginning of the PGDPHM course to avoid response bias.

#### Inclusion and exclusion criteria

Among the PGDPHM participants, nearly 90% were government doctors and the rest were self-sponsored candidates. As the study was aimed at assessing the factors of motivation among government doctors, the responses from self-sponsored candidates were not included in the study. All the government MOs who took the PGDPHM programme and wanted to be part of the study were included. All the government doctors provided responses to the questionnaire, making the group response rate 100%.

The study excluded self-sponsored participants for two reasons. First, the primary objective of the study was to assess driving factors of motivation for only MOs working within government health-care centres. Second, the number of doctors from private hospitals was too small (*N* = 10) to make any comparisons with government MOs.

#### Study areas

The study included MOs from Gujarat, MP and Orissa. All the three states suffer from inadequate number of health centres that include subcentres (SCs), PHCs and CHCs. Furthermore, these states also suffer from shortages of health-care workers (in particular the MOs in Class I and Class II categories) (see Table 2 for details). Table 2 describes the vacant positions of MOs at PHCs and specialists MOs at CHCs. It is important to note that vacant positions (V) are calculated after subtracting in-place (P) MOs from the sanctioned posts (S), given by (S − P). However, the sanctioned positions sometimes do not reflect the actual shortfall of MOs, as the sanctioned positions are sometimes less than what is required by the health system based on population or Indian Public Health Standards norms. Hence, the actual requirement (R) may be even higher and the shortfall of MOs even greater, which are not reflected in Table 2. For example, though the vacancy rates for Class I MOs is 77%, 51% and 42% for Gujarat, MP and Orissa, respectively, the shortfalls for specialists is as high as 93% for Gujarat, 82% for MP and nearly 50% for Orissa [6].

**Table 2:** **Vacant positions of medical officers in the three states studied**^a^

	**Gujarat**			**Madhya Pradesh**			**Orissa**		
**MOs**	**Sanctioned posts**	**In position**	**Vacant, *n *(%)**	**Sanctioned posts**	**In position**	**Vacant, *n *(%)**	**Sanctioned posts**	**In position**	**Vacant, *n *(%)**
PHC MO	1096	837	259 (24%)	1155	541	614 (53%)	1396	1074	205 (15%)
Specialists at CHC	346	79	267 (77%)	502	245	257 (51%)	812	469	343 (42%)

^a^Source- RHS Bulletin 2010 [35].

#### Sample size determination

The total sample size for the study was 92, which is an adequate sample size because 30 or more gives a reasonable approximation [36] for using large sample approximations to the distribution of the test statistics that were used in our data analysis [37,38]. Because the data were in the form of ranks, sample size determination demanded finding the distribution of the ranks under specific alternatives, which were not explicitly available. Therefore, we used a different route. Following Conover [38] and Conover and Iman [39], we assumed that ranks or, more specifically, rank transforms are the values of the variable under study [38,39]. The sample size necessary for finding a difference in population means that equal to M_D_ with power of 1 − −β at level α for a paired *t*-test is given by ${\left(\frac{\left(Z_{\alpha }+Z_{\beta }\right)\sigma_{D}}{M_{D}}\right)}^{2}$, where *Z*_α_ and *Z*_β_ represent, respectively, the upper 100 α and 100 β percentiles of standard normal distribution and σ_D_ is the population standard deviation (SD) of the difference of ranks. Taking α = 0.05, β = 0.2 and σ_D_ = observed SD of the difference of ranks, the minimum sample size required to find a difference M_D_ with power of at least 80% at the 5% level of significance is equal to 4.97 $\sigma_{D}^{2}M_{D}^{2}$. In our current context, an estimate of σ_D_ for the combined data is 17.21. Thus a sample size of 92 is enough to detect a mean difference M_D_ = 4 or more between mean rank scores of intrinsic and extrinsic factors in the population at the 5% significance level with power of at least 80%.

### Study instrument

Following the framework of Herzberg’s two-factor model with some modifications based on a pretest, we provided a questionnaire (developed by Pareek [40]) to each participant. The questionnaire listed seven motivational factors and seven hygiene factors in random order and asked the respondents to rank the factors according to their perceived importance in the current job. The study instrument captured most of the important factors that may lead to MOs’ job-related satisfaction or dissatisfaction. The study instrument has a high reliability of 0.8829. Also, the factor analysis done on the instrument partially validates the two-factor classification, and studies have shown high correlation of 0.87 and 0.99 between intrinsic and extrinsic factors [40].

The study instrument was self-administered, and the respondents were asked the following question: “Rank the fourteen factors according to its perceived importance that motivate you in you in your current job. Rank 1 should be given to the factor that you feel to be the most important and rank 14 to the factor that is perceived to be the least important.” The seven hygiene factors listed were job security, adequate salary, fringe benefits, comfortable working conditions, sound organization policy and practices, considerate and sympathetic supervisor, and restricted working hours. The seven motivational factors were opportunities for promotion, interesting work, respect and recognition, responsibilities and independence, doing something worthwhile, technically competent supervisor, and pay according to ability and competence.

### Data analysis

As discussed in the Introduction, we want to emphasize that the data we collected based on ranks are RPs, which represent the actual choices made in the market in contrast to SPs, which represent choices in a hypothetical context [22]. Thus RP data are more reliable. We carried out the analysis based on the ranks instead of converting it into multiple binary observations and then using discrete choice models [22,23]. The reasons for using this analytical method were twofold. First, use of discrete choice models would make sense if, along with the data on ranking preferences, we had data on the psychographic and demographic characteristics of the MOs. Second, discrete choice formulation is more useful for SP data to control for its unreliability [24].

The data analysis was carried out using SPSS statistical software [41]. Kendall’s coefficient of concordance (W), which is equivalent to the more well-known Friedman test *χ*^2^ statistic for assessing the equality of treatments in a randomized, complete block design [42,43], was computed to see if the rankings assigned by the respondents showed any statistically significant disagreement [37]. Next, we studied the characteristics of the distributions of ranks of different factors by creating box plots [44], as well as using standard descriptive statistics, to come up with an overall ranking of the factors as perceived by the participants. Study of these distributions also helped us understand the ways by which the participants ranked the factors. Finally, we carried out a test of significance to see whether the MOs as a whole were motivated more by intrinsic factors or by extrinsic factors. The overall data analysis was guided by the three main questions that follow.

1. *Testing agreement:* Did the respondents exhibit collective agreement in assigning ranks to the factors with respect to their perceived importance?

To address this question, we computed the coefficient of concordance, W [37], and carried out a right-tailed test to test the null hypothesis that there was no agreement among the rankers (i.e., the participants) against the alternative hypothesis that there was agreement. To explain the testing procedure, we suppose *m* participants (92) are ranking *n* factors (14). To make the illustration simple without loss of generality, we consider *m* = 3 and *n* = 4. In cases where the participants are in complete agreement, the ranks assigned to all three factors would be the same for all four participants. Assuming that there is no tie which was the case in our data set, without loss of generality we assumed that each participant assigned rank *i* to factor F_i_, *i* = 1, 2, 3. The data example are shown in Table 3.

**Table 3:** Ranks assigned to the factors

**Participant**	**F**_**1**_	**F**_**2**_	**F**_**3**_
1	1	2	3
2	1	2	3
3	1	2	3
4	1	2	3
Total	4	8	12

Next, we consider the situation in which there is no agreement in the sense that the participants assigned ranks at random. In other words, the participants randomly assigned one of the six possible arrangements of the ranks {1, 2, 3}. In such a case, the total of ranks corresponding to each factor is expected to be the same. Because the sum total of all ranks assigned by all participants is 24 (4 + 8 + 12), it is equally divided between the three factors. Thus the total rank score for each factor is 8.

If the participants assigned ranks truly at random, the most unlikely event that could be observed would be complete agreement among the participants. In that case, the deviation of the total rank score for each factor from its expected total rank score would be −4 (4–8), 0 (4–4), 4 (8–4), and the sum of squares of the deviations, say, S, would be 32 ((−4)^2^ + (0)^2^ + (4)^2^), which is incidentally its maximum value. On the other hand, it attains the minimum value 0 when the ranks are assigned completely at random. These are the two extreme situations. In general, for a set of observed ranks, S attains a value between 0 and 32. The coefficient of concordance, W, is then defined as S divided by 32, and W takes a value between 0 and 1. It takes the value 1 in cases of complete agreement and 0 in cases of no agreement. In general, for *m* participants and *n* factors following the same argument described above, one can show that $W=\frac{12S}{m^{2}\left(n^{3}-n\right)}$. Under the hypothesis of no agreement, *χ*^2^ = *m*(*n* − 1)*W* has approximately a *χ*^2^ distribution with (n − 1) *df* when *m* is large. The hypothesis of no agreement is rejected at level α if the observed value of *χ*^2^ exceeds $\chi_{n-1,\alpha }^{2}$ (the upper 100 α percentile point of *χ*^2^ distribution with (n − 1) *df*). Otherwise, we would fail to reject the hypothesis of no agreement. Table 4 shows mean values for the 14 motivation factors while Table 5 shows the the observed values of W based on the data for the three states separately as well as for the combined states. In all cases, the *P*-values are approximately zero. Thus our data analysis clearly indicates strong agreement among the participants (see Table 5).

**Table 4:** Mean ± SD and quartiles of ranks for the 14 factors

**Dimension**	**Mean**	**SD**	**Quartiles**		
			**First**	**Second**	**Third**
Intrinsic factors					
Opportunities for promotion	6.47	2.76	5	6	9
Interesting work	4.87	3.25	2	4	7
Respect and recognition	4.91	3.01	4	4.5	7
Responsibility and independence	5.75	3.43	2	5.5	8
Doing something worthwhile	7.28	3.95	4	7	11
Technically competent supervisor	9.48	2.81	8	10	11.75
Pay according to ability and Competence	9.66	3.80	7	10	13
Extrinsic factors					
Job security	4.29	3.84	1	3	7
Adequate salary	5.11	3.81	2	4	7.75
Fringe benefits	9.35	3.30	7	10	12
Comfortable working conditions	6.92	3.43	4	6	9
Sound organizational policies	9.28	3.10	7	10	12
Considerate and sympathetic supervisor	10.24	2.98	9	11	12.75
Restricted hours of work	11.33	3.25	10	13	14

**Table 5:** **Observed values of W and *χ***^**2 **^**for the three states separately and combined as a group**

**State**	**Observed value of W**	**Observed *χ***^**2 **^**value**	***P*-value**
Gujarat	0.34	229.84	9.65912E-42
Madhya Pradesh	0.33	94.38	2.01889E-14
Orissa	0.38	88.92	2.24773E-13
Combined	0.32	382.72	9.9304E-74

2. *Overall ranking:* Because the data exhibited strong evidence of agreement among the participants, the next question that arose was, Could we assign an overall rank to these factors? (See Tables 4 and 6).

**Table 6:** Average and ordered ranks for various factors of motivation

**Ordered rank**	**Motivational factor**	**Intrinsic/extrinsic**	**Average rank**
1	Job security	Extrinsic	4.29
2	Interesting work	Intrinsic	4.87
3	Respect and recognition	Intrinsic	4.91
4	Adequate salary	Extrinsic	5.11
5	Responsibility and independence	Intrinsic	5.75
6	Opportunities for promotion	Intrinsic	6.47
7	Comfortable working conditions	Extrinsic	6.92
8	Doing something worthwhile	Intrinsic	7.28
9	Sound organizational policy	Extrinsic	9.28
10	Fringe benefits	Extrinsic	9.35
11	Technically competent supervisor	Intrinsic	9.48
12	Pay according to ability and competence	Intrinsic	9.66
13	Considerate and sympathetic supervisor	Extrinsic	10.24
14	Restricted hours of work	Extrinsic	11.33

As a proxy for overall rank, average rank across the respondents is used for each factor. The underlying principle is the lesser the average rank of a factor, more preferred it is. This, however, makes sense if the SDs of the ranks across the factors can be assumed to be the same. Otherwise, a better method would be to rank the factors by the ratio of average rank to their SDs. Because the SDs across the factors do not seem to be significantly different (see table and Results section for details), we use average rank as a proxy for overall rank of a factor (see Table 6).

3. *Intrinsic or extrinsic*: Are the participants more intrinsically or extrinsically motivated?

Finally, to address the research question whether participants are more intrinsically or extrinsically motivated, we carried out two-sample Kolmogorov-Smirnov tests [35,38] for each group (i.e., state) separately and for the combined group to compare the distributions of intrinsic and extrinsic scores. The intrinsic (extrinsic) score of an individual is the sum of ranks assigned by him or her to the intrinsic (extrinsic) items. This is a nonparametric test that does not need any distributional assumptions for carrying out the test of significance (see Table 7).

**Table 7:** ***P*-values of Kolmogorov-Smirnov test comparing intrinsic and extrinsic score distributions**

**State**	**Kolmogorov-Smirnov Z**	**Asymptotic *P*-value (two-tailed)**
Gujarat	1.961	0.001
Madhya Pradesh	1.658	0.008
Orissa	0.833	0.491
Three states combined	2.507	0

## Results

The results of the study are arranged based on the three research questions addressed above.

1. Did the respondents exhibit collective agreement in assigning ranks to the factors with respect to their perceived importance?

The study results indicate strong statistical agreement among the ranks assigned by the participants. This is based on observed values of W based on the data for the three states separately as well as for the combined states. In all cases, the *P*-values are approximately zero.

2. Because the data exhibited strong evidence of agreement among the participants, the next question that arose was, Could we assign an overall rank to these factors?

**Figure 1:**
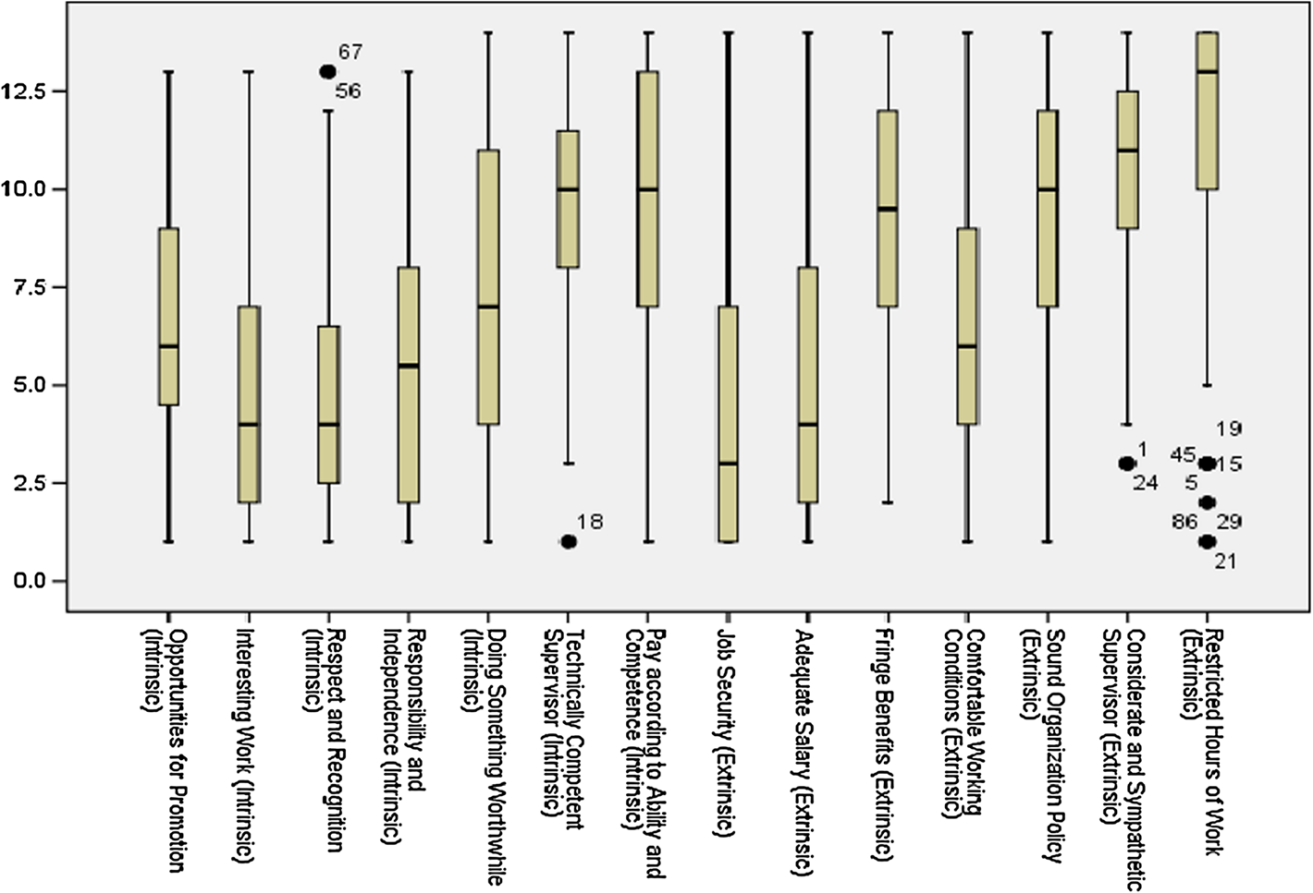
Box plots for the distributions of ranks of the 14 factors of motivation across the participants.

**Figure 2:**
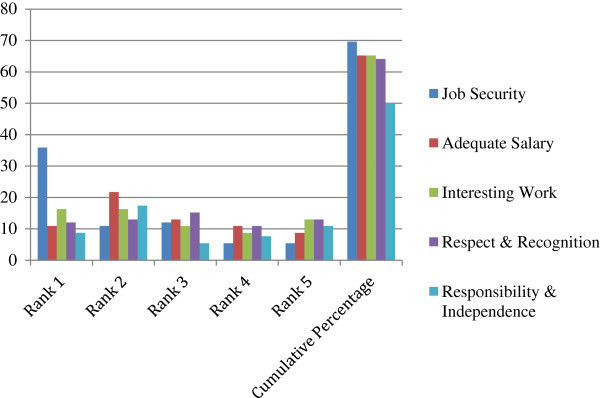
Bar diagram showing the percentage of participants ranking the top five factors of motivation.

**Figure 3:**
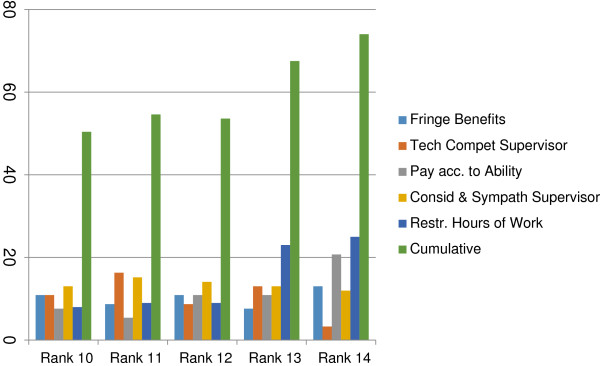
Bar diagram showing the percentage of participants ranking the bottom six factors of motivation.

Because the SDs across the factors do not seem to be significantly different (see Table 6), we use average rank as a proxy for the overall rank of a factor. In an ideal case, it would be expected that there would be a strong consensus among the participants in ranking a few factors at the top and a few at the bottom, but the strength of agreement would be expected to be less for factors lying in the middle. However, Table 4 shows that the strength of agreement of the participants across the factors measured by SD is more or less same. This could be explained by the data in Table 6 and the box plot exhibiting the distribution of ranks for each factor (Figure 1). For example, even if job security is found to be the most preferred factor, approximately 25% participants assign ranks 7 or more to it (see Table 6). In other words, for at least 25% of participants, it is not high on their preference list as a factor of motivation. On the other hand, though, restricted hours of working is least preferred by a majority of the participants, there were a few participants (shown as bold dots, outliers) who assigned a very low rank to it, in other words showing a high preference for it as a factor of motivation. Clearly, each factor receives high as well as low rankings, but those at the top and at the bottom of the preference list received low and high ranks, respectively, from the majority of the participants. Figure 2 shows that about 36% assigned rank 1, 12% each ranked 2 and 3, 5.4% each ranked 4 and 5 to job security, and the cumulative percentage of participants assigning ranks less than or equal to 5 for job security was about 80%. Figure 2 also shows the percentages for four other factors at the top. For the bottom five factors, Figure 3 shows a similar pattern, with a majority of the participants exhibiting consensus in assigning high ranks.

3. Are participants more intrinsically or extrinsically motivated?

The *P*-values are given in Table 7. It is evident that for Gujarat and MP as well as for the combined group, the distributions of intrinsic and extrinsic scores are different at both the 5% and 1% levels of significance. In fact, the *P*-values show that the strength of rejection of the null hypothesis of equality of distributions is much stronger than that reflected by the 1% level of significance. For Orissa, however, the *P*-value shows strongly that the distributions are not significantly different. Also, the empirical distribution function of the extrinsic scores is clearly located to the right of that of the intrinsic scores for Gujarat, MP, and the combined group. In other words, the participants in these cases tended to assign lower ranks to the intrinsic items compared to the extrinsic items. Thus the data analysis shows that the participants are plausibly more intrinsically motivated.

Thus we found job security to be the most preferred factor of motivation, closely followed by interesting work and respect and recognition. Among the five most preferred factors, three are intrinsic or job content factors (interesting work, respect and recognition and responsibilities and independence). It clearly exhibits the perceived importance of intrinsic factors to the MOs beyond monetary benefit and job security. The least preferred factor is restricted working hours, with an average rank of 11.33. MOs seem to be least concerned about it. There may be two reasons. Either they are prepared for it knowing the kind of service they are in or the work environment provides enough room to balance their professional and personal lives.

## Discussion

The key findings of the study are a statistically significant (*P* < 0.01) agreement exhibited by the participants in ranking the factors of motivation with respect to perceived importance and that the overall importance placed on intrinsic factors is significantly more than that placed on the extrinsic factors. Hence it can be concluded that MOs’ motivation to continue with the current job is driven by both intrinsic and extrinsic factors. However, the importance placed by MOs on each factor of motivation is different. For example, the study results indicate that job security is the most preferred factor of motivation, closely followed by interesting work and respect and recognition. Among the top five preferred factors, three are intrinsic factors or job content factors, indicating a great importance given by MOs to factors beyond monetary benefit and job security. The study results also indicate that MOs are motivated more by intrinsic needs that go beyond the general understanding that MOs are motivated only by extrinsic factors. Looking at the study results, we can conclude that more importance is significantly placed on intrinsic factors than on extrinsic factors. This has very interesting policy implications, such as prioritizing the identification and placement of intrinsically motivated MOs over modifying extrinsic factors such as work conditions.

To consolidate our study results, we now invoke the findings of similar studies done in the past in India and elsewhere. Specifically, we discuss why participants identify some factors as important. Is there any rationale or past evidence?

### Job security

The participants considered job security to be the most important factor. In India, among all kinds of jobs, government employment is most secure. Once one enters into the system, the job is permanent and the chance of losing the job is practically negligible. The monetary benefits, such as pension and gratuity, along with nonmonetary benefits such as less workload, are some of the important factors that attract doctors to join and continue in government service. A recent study on government doctors working in rural areas confirms such findings [45]. Similarly, researchers in a study on rural health-care workers of North Vietnam reported that job stability and income were found to be the major motivating factors [13].

### Interesting work

According to our study results, one of the most important driving factors of motivation for MOs is interesting work. However, the meaning of the term *interesting work* is subjective. Just as each individual has different motivations to work, each individual may also have his or her own reasons for finding the work interesting. For some, it may be the challenges in work; for others, it may be the opportunities to use their knowledge and skills; others may find sheer professional and scientific interests rewarding; and for yet others, it may be the ability to influence health outcomes. A recent study in the Indian context suggested that MOs were inspired by unique challenges to influence health outcomes in rural areas [45]. The same study also found that feeling of ‘personal fulfilment’, ‘usefulness of practicing in rural areas’ and ‘professional and scientific interest’ were important factors for work motivation [45,46].

### Respect and recognition

Our study reports ‘respect and recognition’ as an important factor of motivation. This finding is corroborated by those of several other recent studies in the health-care sector. For example, a study of health-care workers carried out in North Vietnam and Mali reported that ‘recognition, responsibility and training’ were the main motivators at work [7,47]. There is further evidence from a study done in Bangladesh with community health workers (CHWs) that reported ‘recognition’ as one of the most important reasons for CHWs to continue with the job [48]. Although it is true that the roles of CHWs are very different from those of MOs and that the two cannot be compared, the main idea of drawing inferences from the Bangladesh study is to demonstrate the importance of ‘recognition’ as an important intrinsic factor of motivation for health-care workers.

### Adequate salary

‘Adequate salary’ is one of the important hygiene factors agreed upon by the participants for their work motivation. The existing evidence in research about monetary incentives as a positive motivator is mixed and inconclusive. Empirical evidence indicates that though money plays an important role in health-care worker motivation, it is not the only reason for health-care worker shortage [49,50]. Similar research done outside India indicates that monetary incentives play only a limited role in motivation, especially when the increased monetary rewards are nominal [9].

Researchers in a recent study of health-care workers in Pacific and Asian countries reported that financial incentives such as high salary is an important motivating factor, especially in countries where government salaries are not sufficient to meet the basic needs of health-care workers [7]. Similarly, studies done in Fiji, Cambodia and North Vietnam have identified low salaries as a common reason for job dissatisfaction among health-care workers [13,49]. A study done in Peru suggests that absence of adequate salary is an important reason why doctors engage in dual practice [51]. A similar study done in Bangladesh indicates the importance of higher pay among doctors in government PHCs to be sufficient for them to give up dual practice (i.e., having a private practice at the in addition to their government service duties) [47,52].

### Opportunities for promotion

The study respondents also identified opportunities for promotion as an important motivating factor. There are many studies that support this finding. For example, limited opportunities for professional development have been found to be a reason for dissatisfaction with jobs in Tonga [11,41]. Similarly, a study done in Fiji, Samoa and Tonga found a limited scope for upgrading qualifications, lack of promotion and career prospects and career structure as reasons for dissatisfaction and migration among doctors [11,42,47,48]. Also, researchers in a recent study done in India reported that one of the reasons why MOs are likely to continue in government service is seeking higher positions in the government [7,46].

### Comfortable and adequate working conditions

The overall ranking MOs assigned to this factor was 7, indicating moderate importance. Evidence shows that inadequate working conditions and facilities, as well as shortages of drugs and equipment, in Fiji, Samoa, Tonga, Cambodia and Pakistan contribute to dissatisfaction of health-care workers with their current jobs [49,53,54]. A study done in India also found that MOs practicing in rural areas are unhappy with the support and facilities available at their health-care centres [45]. Research indicates that inadequate support, supervision and management may lead to reduced work motivation [13,49,55].

### Doing something worthwhile

Although the meaning of the term *doing something worthwhile* is subjective, a research study done in India identified ‘personal values of service to poor’ and ‘selflessness’ as important motivating factors for MOs to work in rural health-care services [45]. Research in the area of organizational behaviour indicates that ‘superordination goals’ contribute to role efficacy among workers. Such goals are met when employees have a feeling that they are contributing something worthwhile to the organization or to larger sections of society [34].

### Sound organizational policies

The findings of the present study indicate that sound organizational and departmental policies are of moderate importance to MOs. There is evidence that poor practices related to posting and transfer are causes of low morale, inadequate geographical distribution and migration of MOs [56]. Similarly, the findings of studies of the water and irrigation sector in India suggest the existence of a parallel system of postings and transfers that leads to corruption [57,58].

Empirical and anecdotal evidence indicates that human resources (HR) policies, rules and practices related to recruitment, placement and transfer in the India health sector are mostly inefficient and nontransparent [59]. The absence of transparent policies and efficient systems can sometimes act as barrier to assigning MOs to the right places or health-care facilities. Therefore, there is a great need for clearer and more transparent HR policies.

## Recommendations

As our study results indicate the importance of both intrinsic and extrinsic factors, we recommend a bundle of strategies that are a mix of both intrinsic and extrinsic factors. In this section, we focus on the most important factors of motivation identified by MOs.

First, from a health-care system point of view, it is very important that MOs be provided with some kind job security and monetary benefits. MOs serving in rural areas must be provided with an extra monetary allowance. Such an allowance can be paid in the form of hardship allowance. Care must be taken to fix the rate of hardship allowance so that MOs find it attractive enough to work in rural areas. Although there are already many states in India that are paying hardship allowances to MOs to encourage them to serve in rural areas, there is wide variation in the amount of the hardship allowance paid by different states [8].

The state health departments must also pay close attention to make the work of MOs more interesting. For example, this could be done by introducing more challenges in the current job. In order to provide recognition, the health department needs to create a system of rewarding and recognizing good performers. The system should have a process in place to continuously and periodically monitor the performances of MOs. Rather than waiting for and rewarding the MOs for big achievements, small achievements should be recognized and rewarded in monetary and nonmonetary terms. Such practice could lead to self-motivation and higher satisfaction [60]. However, it is recommended that the overall incentive system, both monetary and nonmonetary, should be performance-based.

The state health departments must make efforts to develop MOs in their current roles through appropriate training to upgrade their knowledge and enhance their skills. Creating an efficient system for placing MOs in appropriate positions is also very important to avoid role monotony and stagnation and thus increase job satisfaction and motivation.

## Limitations and strengths

The study results are more indicative than representative of the MO fraternity and should not be completely generalized to represent the driving factors of work motivation for MOs in other geographical areas for two reasons. (1) The present research was based on a limited number of MOs in three states. However, we argue that the study has interesting findings that are corroborated by findings of similar studies done within and outside India. Hence, the study results have interesting management implications related to motivation that are relevant to India and other countries. (2) Our present study was confined to 14 factors of motivation (based on the Herzberg theory of motivation). It does not include a few other important factors of motivation, such as educational facilities and prospects related to child care, logistics problems, and workload and safety issues that have been reported to be important in other studies [13,47,54]. Furthermore, the present study provides a view of only what is regarded as important by MOs who are currently working. In no way do the study results indicate that the factors ranked high (from the top) are met in the MOs’ current jobs or that the factors ranked low (from the bottom) are not met. Finally, the study is only partially based on the Herzberg theory of motivation.

Despite the limitations of the study, it is important for the following reasons. (1) To the best of our knowledge, this study is one of the very few (in India and elsewhere) related to motivation of MOs that is built on a ranking exercise based on a theory of work motivation (proposed by Herzberg) to identify factors of motivation for in-service MOs to continue with their present jobs. (2) The findings of the study have interesting policy implications. (a) The participants placed equal, if not more, importance on intrinsic factors compared to the extrinsic factors. Contrary to common belief, on average, MOs assign more importance to intrinsic factors than on extrinsic factors. (b) Despite the fact that the respondents are from different states representing different health-care facilities (PHCs, CHCs and DHs), the respondents exhibit strong agreement in ranking the factors. From the policymaker’s perspective, this makes it simpler to identify and devise management strategies to address the critical issue of shortage of MOs. The study also offers a useful lesson that the Herzberg theory cannot be applied as a blanket approach to study motivation and that intrinsic and extrinsic factors may be the same for some and very different for others. Hence, such factors cannot always be treated as opposites of one another. Finally, although the sample size of the study was 92, it still represented more than 25 districts in India, which is a fairly large number of districts and covers a large geographical area.

## Conclusion

There is a dearth of evidence in the literature about which strategies work the best to address the shortage of MOs. In general, most of the public health sector interventions have focused on hygiene factors of motivation to address the problem of health-care workforce shortages. These factors typically include higher pay and allowances, better working and living conditions, compulsory rural service, postgraduate allowances and hardship allowances. The study results indicate that addressing the motivation of MOs requires a bundle of strategies (a mix of both hygiene and factors of motivation) to respond to the motivational needs of MOs. Therefore, we strongly recommend that the Indian national and state health departments, policymakers and reformers devise management strategies that address both intrinsic and extrinsic factors of motivation. Although Herzberg’s theory provides a useful framework with which to study factors of motivation, care must be taken to apply this theory to study work motivation based on different health-care settings.

## Competing interest

The authors declare that they have no competing interests.

## Authors’ contributions

BP conceived the study, selected the appropriate instrument for the study and collected the data. The data analysis was conceived and written by TB. BP wrote the first draft of the manuscript, and TB’s suggestions led to substantial improvement in the presentation of the material. BP is guarantor of the paper. Both authors read and approved the final manuscript.
